# A case of tracheal injury with cricoid fracture due to blunt laryngeal trauma

**DOI:** 10.1016/j.tcr.2026.101376

**Published:** 2026-05-21

**Authors:** Eri Uemura, Shimon Murahashi, Shuhei Yamano, Hiroo Izumino, Takamitsu Inokuma, Goro Tajima, Kazunori Yamashita, Sumie Takashima, Yoshihiko Kumai, Osamu Tasaki

**Affiliations:** a Department of Emergency Medicine, Graduate School of Biomedical Sciences, Nagasaki University, 1-12-4 Sakamoto, Nagasaki, 852-8523, Japan; b Acute and Critical Care Center, Nagasaki University Hospital, 1-7-1 Sakamoto, Nagasaki, 852-8501, Japan; c Department of Otolaryngology-Head and Neck Surgery, Nagasaki University Graduate School of Biomedical Sciences, 1-7-1 Sakamoto, Nagasaki, 852-8501, Japan; d Department of Neurointensive Care, Neuro ICU and Coma Science Center, Toda Medical Group Asaka Medical Center, Saitama, Japan

**Keywords:** Cricoid cartilage, Fracture, Tracheal injury, Traumatic blunt injury, Neck, Airway management

## Abstract

**Background:**

Laryngeal injuries, especially cricoid cartilage fractures, are rare but may present with airway emergencies, and appropriate airway management is vital.

**Case presentation:**

A 24-year-old man admitted to the emergency room with isolated blunt cervical trauma after a motorcycle accident. The patient presented dysphonia, stridor, hemoptysis, cervical subcutaneous emphysema, and increasing respiratory distress. Oral intubation was initially successful, however, worsening subcutaneous emphysema and loss of effective ventilation suggested airway disruption. Emergency cricothyrotomy was performed, but persistent air leakage from the incision site prompted bronchoscopy, which revealed a 7-mm tracheal laceration on the right side of the larynx. The patient was reintubated under bronchoscopy. Computed tomography showed a cricoid cartilage fracture. Tracheostomy and complete repair were performed 2 days after the injury. The cricoid cartilage was fractured fully circumferentially, with the anterior part deviated toward the caudal side, and its deepest part had perforated the right side of the trachea. The cricoid cartilage was repaired, and the tracheal perforation was dilated to create a laryngo-cutaneous fistula. Persistent hoarseness after fistula closure was associated with scar formation of the left vocal fold and arytenoid cartilage dislocation. Although symptoms gradually improved with voice rehabilitation, increased vocal demand after a job change led to thyroplasty 2 years after injury.

**Conclusion:**

Cricoid cartilage fractures associated with blunt laryngeal injuries may cause tracheal injury. This case highlights the challenges of managing concurrent cricoid fracture and tracheal injury, where the surgical airway site coincided with the traumatic defect. Rapid decision-making balancing exploration and stabilization was critical.

## Highlights

• Cricoid fractures may complicate airway management in laryngeal trauma.
• In this case, tracheal injury at the cricothyrotomy site impaired ventilation, highlighting the need for flexible airway strategies and prompt identification of the injured site.

## Introduction

The larynx is anatomically located such that it is difficult to damage. Laryngeal trauma is rare, accounting for less than 1% of blunt trauma cases, and is seen only in 1 in 30,000 patients treated at tertiary care trauma centers [1], [2]. Especially, cricoid cartilage fractures are rare and reportedly account for less than 5% of all laryngeal trauma cases [3]. However, cricoid cartilage fractures can cause upper airway obstruction, and proper airway management is vital.

## Case report

A 24-year-old male was admitted to the emergency room with cervical trauma after a motorcycle accident. The patient struck his anterior neck against the motorcycle handlebar during deceleration, causing direct compression of the larynx against the cervical spine. Immediately after the injury, he complained of neck pain and dysphonia. The patient was conscious and oriented (Glasgow Coma Scale of 15), hemodynamically stable, and was able to walk to the ambulance on his own. However, on the way to our hospital, he developed dyspnea and stridor. On hospital arrival, he was unable to lie supine and was placed in a forward-leaning position on a stretcher. The patient had stridor, hemoptysis, and labored breathing. His physical examination revealed neck bruising over the thyroid cartilage, crepitus extending to the supraclavicular region, and stridor on auscultation. The trachea was slightly shifted to the right.

We determined that the patient was having an airway emergency, and oral endotracheal intubation was achieved using a McGrath laryngoscope with an otolaryngologist standing by to secure a surgical airway. Intubation was performed in a semi-Fowler position with neck stabilization. Sedation was achieved with propofol 4 mg and fentanyl 50 μg, avoiding muscle relaxants due to airway compromise risk. Although the intubation tube passed through the glottis smoothly, there was no fogging of the intubation tube and no movement of the reservoir bag in response to breathing. The patient's SpO_2_ decreased, subcutaneous emphysema increased, and the endotracheal tube removed. Subsequently, a cricothyrotomy was performed by the otolaryngologist. After the cricothyrotomy, the patient began coughing, with blood gushing from the tube, and his subcutaneous emphysema was increasing. Tracheal injury was observed on the right side of the incision, which was then compressed with gauze. Following this, air leakage and the gushing of blood stopped, and the patient was able to ventilate. Bronchoscopy was performed while compression was continually applied to the cricothyrotomy site.

**Fig. 1:**
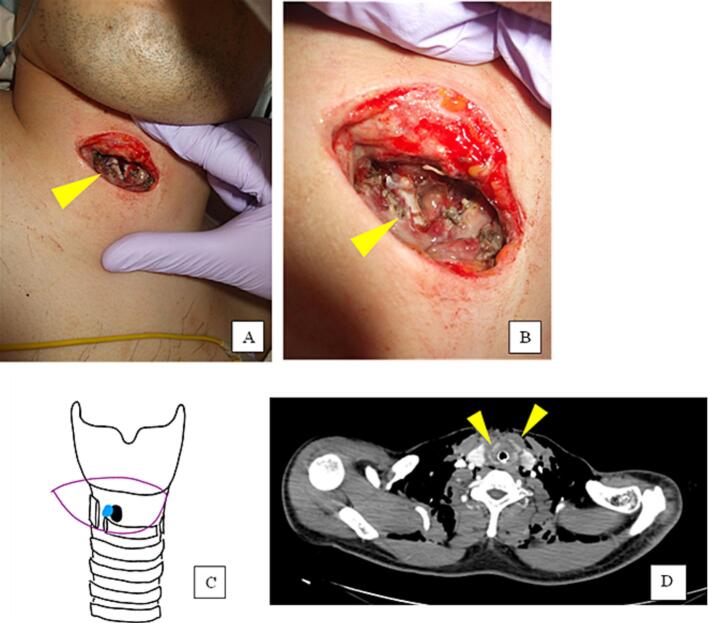
The cricothyrotomy site after oral tracheal intubation. (A) A general view of the neck shows that the neck is swollen due to subcutaneous emphysema. (B) Proximal view of the injured area. The fractured cricothyroid cartilage is indicated by the arrowheads. (C) Schema of internal anatomy. The blue hole is the tracheal injury site and the black hole is cricothyrotomy hole. (D) Computed tomography images show fractures of the cricoid cartilage as indicated by the yellow arrowheads, with surrounding subcutaneous emphysema.

The bronchoscopy revealed a 7-mm-large laceration on the right side of the trachea below the vocal cords. The vocal cords were found to be edematous and bilaterally fixed in the midline. The patient was reintubated orally under bronchoscopy, and the tip of the endotracheal tube was fixed distal to the injury, 2 cm cephalad of the tracheal bifurcation, which stabilized the patient's respiratory status. After oral endotracheal intubation, examination of the cricothyrotomy site revealed a fractured cricoid cartilage, as shown in Fig. 1A, B and C.

A computed tomography scan of the neck was then performed that revealed a fracture of the cricoid cartilage and surrounding subcutaneous emphysema (Fig. 1D). The patient was also found to have bilateral pneumothorax and a displaced right radial forearm fracture. A thoracic drain was inserted for the left pneumothorax.

**Fig. 2:**
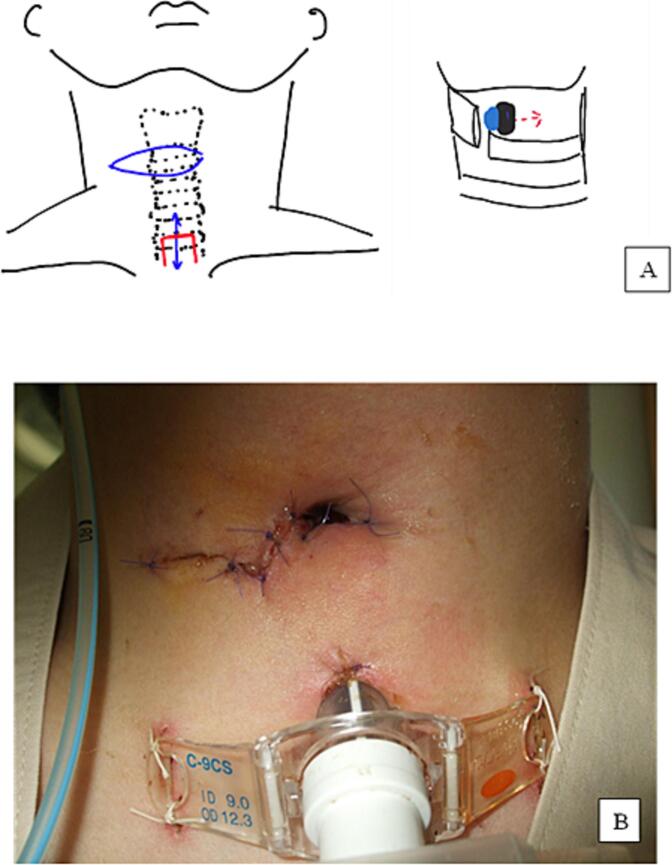
Schema of the injury and operation anatomy. (A) The blue line is the skin incision line of the cricothyrotomy. The blue hole is the tracheal injury site and the black hole is cricothyrotomy hole. The anterior part of the cricoid cartilage was deviated caudally, and a perforation was seen on the right side of the trachea. The cricoid cartilage was repaired, the tracheal perforation was dilated to the left side (A: right panel of red dashed line), and the laryngo-cutaneous fistula was created (B yellow oval). A tracheostomy was performed through a median longitudinal incision (A: left panel, blue solid line) below the cricothyrotomy site. The tracheostomy was placed between the 3rd and 4th tracheal rings (A: left panel, red line).

Tracheostomy and cricoid cartilage fixation were performed on hospital day 3(Fig. 2). The tracheostomy was performed through a median longitudinal incision made below the cricothyrotomy site and was placed between the 3rd and 4th tracheal rings. The cricoid cartilage showed a circumferential fracture line all around, and the anterior portion of the cricoid cartilage deviated posterolaterally. Perforation was observed on the right side of the trachea as shown in Fig. 2. The vocal cords were identified 4 cm cephalad of the tracheal perforation. The vocal cords were strongly edematous but were preserved morphologically.

The cricoid cartilage was repaired, the tracheal perforation was dilated, a laryngo-cutaneous fistula was created, and the surgery was completed. The area around the tracheal perforation was contused, which suggested that the site of cricothyrotomy and the injured area were in the same location.

After tracheostomy, the patient was weaned from the ventilator, and the chest drain was removed on the second postoperative day (Hospital day 5). The patient was switched to a speech cannula on hospital day 16, and he started drinking on hospital day 17. The tracheostomy hole was closed on hospital day 19, and the patient began to take food orally. He was discharged home on hospital day 23 and returned to work on hospital day 36.

The fracture of the distal part of the right radius was treated with external fixation on the same day of his presentation. Hospital day 6, the patient underwent plate fixation of the distal end fracture of the right radius. The postoperative course was uneventful, and the patient returned to work on postoperative day 17.

The laryngo-cutaneous fistula was closed under local anesthesia 3 months after the injury. Following closure of the laryngo-cutaneous fistula, hoarseness persisted. Flexible laryngoscopy demonstrated scar formation in the posterior left vocal fold with compensatory false vocal fold phonation. Voice rehabilitation was subsequently initiated. 6 months after injury, follow-up laryngoscopy demonstrated resolution of the granulation tissue associated with the pharyngo-cutaneous fistula. Computed tomography performed 10 months after injury revealed dislocation of the left arytenoid cartilage; however, surgical intervention was deferred because the hoarseness gradually improved with rehabilitation. The Voice Handicap Index (VHI) score was 48 approximately 1 year after the injury. Approximately 2 years after the injury, the patient changed occupations, resulting in increased vocal demand. Thyroplasty was subsequently performed. No airway-related complications were observed during follow-up.

## Discussion

In the present case, the patient presented with an airway emergency that had developed during transport, although the patient was initially intact after the injury. The cause of dyspnea in this case was thought to be airway narrowing due to fracture, edema and mucous membrane damage, as well as blood sputum from tracheal perforation. On presentation, the patient was having an airway emergency, endotracheal intubation was attempted. But it was presumed that the intubation tube had deviated into the tracheal perforation site. Although a cricothyrotomy was performed by an otolaryngologist, even when the cuff was inflated, and subcutaneous emphysema was assumed to have increased from the tracheal injury site. Endotracheal intubation was performed again with the aid of a bronchoscope, and the endotracheal tube was placed in the appropriate position.

CT showed a vertical fracture of the cricoid cartilage and a deviation of the cricoid cartilage to the left. These findings suggest that the tracheal perforation was located on the right side of the cricoid cartilage, and the tip of the intubation tube may have penetrated the perforation site at the time of tracheal intubation. Furthermore, the tracheal perforation hole was connected to the incision created by a cricothyrotomy; the tracheal tube used was so small that air leakage occurred.

It should be kept in mind that if the patient is unable to speak at all, as in this case, the laryngeal cartilage may be deviated to a large degree, and oral intubation may be difficult.

Laryngeal injuries are rare, but they may cause airway obstruction [3]. While laryngeal injuries are reported to account for less than 1% of all blunt trauma, cricoid cartilage fractures are reported to account for 5–24% of these injuries [3], [4]. Isolated cricoid fractures may be life-threatening because the cricoid is the only circumferential cartilage protecting the larynx and is essential for stability and integrity of the airway [5]. Hoarseness, subcutaneous emphysema, and tenderness are signs of laryngeal injury [6]. Stridor and hemoptysis/sputum are reported to be associated with severity of injury, and tracheostomy should be considered immediately if the patient cannot be placed in a supine position [1]. Tracheotomy is recommended as a means of securing the airway because tracheal intubation in patients with laryngeal injuries can cause complications in the injured area [3]. However, in patients with airway obstruction or severe respiratory distress, oral tracheal intubation may also be recommended as a means of securing airway [6].

**Fig. 3:**
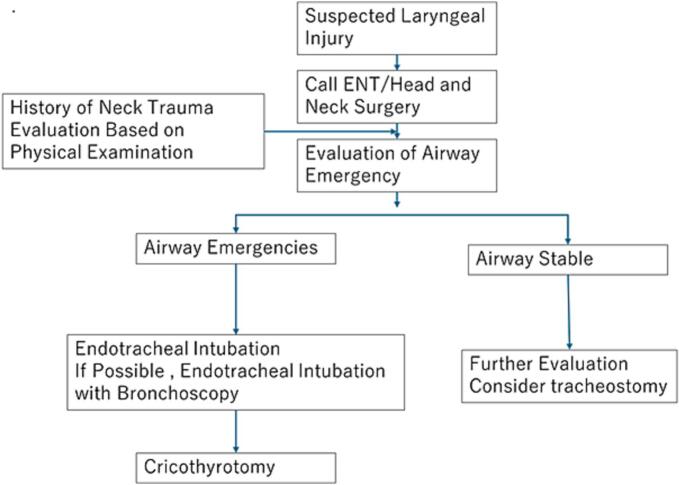
Algorithm for airway management of laryngeal trauma.

If intubation is unsuccessful or conditions for optimal intubation cannot be met, and if tracheotomy is not feasible, cricothyrotomy with subsequent revision should be considered [2]. If the situation permits, bronchoscopy-assisted tracheal intubation, which also allows observation of the larynx and tracheal lumen, has been reported to be safer and preferable [3]. In the present case, there was no time for tracheostomy or bronchoscopy due to rapidly increased emphysema and decreased SpO2. Based on the experience of the present case, we propose an algorithm for airway management of laryngeal trauma in Fig. 3.

## Conclusion

Cricoid cartilage fracture is associated with laryngeal injuries. In the present case, the tracheal injury occurred at the same site as the cricothyrotomy, presumably resulting in impaired airway clearance and ventilation. Airway management of laryngeal injuries requires the preparation of several methods to handle potential complications, and the injured area must be identified as soon as possible.

## CRediT authorship contribution statement

**Eri Uemura:** Writing – original draft, Data curation. **Shimon Murahashi:** Writing – review & editing. **Shuhei Yamano:** Writing – review & editing. **Hiroo Izumino:** Writing – review & editing. **Takamitsu Inokuma:** Writing – review & editing. **Goro Tajima:** Writing – review & editing. **Kazunori Yamashita:** Writing – review & editing. **Sumie Takashima:** Writing – review & editing. **Yoshihiko Kumai:** Writing – review & editing. **Osamu Tasaki:** Writing – review & editing.

## Ethics statement

Approval of the research protocol: N/A.

Informed consent: Informed consent was obtained from the patient.

Registry and the Registration No. of the study/Trial: N/A

Animal Studies: N/A

## Source of funding

None.

## Declaration of competing interest

The authors declare that they have no known competing financial interests or personal relationships that could have appeared to influence the work reported in this paper.
